# Tutor-demonstrated feedback in the mini-clinical evaluation exercise

**DOI:** 10.15694/mep.2020.000130.1

**Published:** 2020-06-23

**Authors:** Madhur Bhattarai, Sean McAleer

**Affiliations:** 1 Chitwan Medical College; 2 Centre for Medical Education

**Keywords:** Assessment, feedback, feedforward, learning by observation, learning for assessment, mini-CEX, PACES, peer-assessment, supervised learning events, teaching for assessment

## Abstract

This article was migrated. The article was marked as recommended.

**Introduction:** The mini-clinical evaluation exercise (mini-CEX) allows for assessment and subsequent feedback across a range of individual clinical cases, although much of what is assessed is left implicit. After the performance by a trainee, further observation of appropriate clinical performance by the tutor provides a strong standard of reference for effective learning.

**Methods:** In each mini-CEX encounter one final year resident clinically assessed an unfamiliar patient in the presence of peers and a tutor. The tutor then demonstrated the appropriate clinical behaviour. Twelve peers rated the performance of the resident before and after the tutor-demonstrated feedback (TDF). The encounters with the first cases by each of the participants were completed one by one and the cycles of encounters with the next cases by each resident were similarly continued.

**Results:** All 13 participants completed six mini-CEX encounters over one year with an overall response rate of 95% for peer-assessment ratings (PARs). There was a total of 1772 PAR forms each with seven parameters and 1772 peer-satisfaction rating (PSR). Reliability coefficients of PARs ranged from .77 to .93. The PARs decreased significantly in each of the six cases after TDF (p<0.001). Peers rated the performance more negatively after the TDF than before the TDF. The mean PSRs, however, increased significantly in each of the six cases after the TDF (p<0.05). The mean observation time of participants’ performance decreased from 26 minutes in the initial two cases to 14.5 in the last two (p<0.001).

**Conclusion:** The feasibility and the positive educational impact of the TDF were shown in our exploratory study. The novel concept of TDF in the mini-CEX as supervised learning events and formative assessment deserves further investigation.

## Introduction

First introduced more than two decades ago by Norcini *et al*. (1995), the mini-Clinical Evaluation Exercise (mini-CEX) has become established as a convenient and effective educational tool. A mini-CEX encounter typically consists of a single faculty member observing a resident while that resident conducts a focused history and physical examination. After asking the resident for a diagnosis and treatment plan, the faculty member rates the resident and provides educational feedback ( Norcini *et al*., 1995). The mini-CEX is now commonly used as part of supervised learning events ( Joint Royal Colleges of Physicians Training Board, 2014) and formative assessment with trainees ( American Board of Internal Medicine, 2020).

Miller and Archer (2010) in their systematic review concluded that though the subjective perception and satisfaction reports of the mini-CEX were positive, there was no evidence that it led to further improvement in the performance of doctors. Other reviews by Kogan *et al*. (2009) and Pelgrim *et al*. (2011) also pointed to the scarcity of supporting evidence of positive educational outcomes relating to medical students and doctors. In a recent meta-analyses Lorwald *et al*. (2018) found some evidence of positive effects on trainee performance and reported two implementation characteristics associated with the educational impact of the mini-CEX: how well different program components are conducted (i.e. quality of the implementation) and the degree to which the program stimulates the interest or holds the attention of participants (i.e. participant responsiveness during implementation). How to maximize the quality of performance and participant responsiveness during implementation of the mini-CEX is a key issue.

The mini-CEX involves observation of what actually happens in clinical practice ( Hawkins *et al*., 2010). Among the various clinical skills that can be observed and assessed during such clinical encounters are professional behaviour, medical interviewing, physical examination, identification of physical signs, communication and counseling, clinical interpretation and judgment, managing patients’ concerns and maintaining their welfare, overall clinical efficiency and concise summarization ( Membership of the Royal Colleges of Physicians of the United Kingdom, 2017; ABIM, 2020). In the mini-CEX, a faculty member rates the trainee and provides feedback. Simply providing information about whether the performance is right or wrong does little to help students correct their errors. Including the correct answer as part of the feedback message is critical ( Larsen, Butler and Roediger III, 2008). van de Ridder *et al*. (2008) define feedback as specific information about a trainee’s observed performance measured against a standard with the intent to improve the trainee’s performance. The standard has to be explicit. An implicit standard is linked to weak feedback and an explicit standard to strong feedback (van de Ridder *et al*., 2008). However in the mini-CEX much of what is assessed is implicit and is up to the discretions of the assessors ( Pelgrim *et al*., 2011).

Health professionals are required to competently perform a wide range of practical skills - from carrying out procedures, to examining patients and effective communication ( Lake and Hamdorf, 2004). A common approach to skills teaching is Peyton’s 4-Steps-Approach ( Lake and Hamdorf, 2004): demonstration (trainer demonstrates at normal speed, without commentary), deconstruction (trainer demonstrates while describing steps), comprehension (trainer demonstrates while learner describes steps) and performance (learner demonstrates while learner describes steps). In the mini-CEX, after the performance of the trainee the tutor can also actually demonstrate on the spot the appropriate clinical behaviour. Such a demonstration by the tutor would mark the explicit standard upon which to base feedback to the trainees about any performance gaps. In any clinical encounter, the trainees should be evaluated and given feedback based on their ability to perform as the faculty member would perform ( MRCP, 2017). The clinical picture and findings of each patient are unique. The demonstration of the appropriate performance in such a situation by the faculty member plays a key role in the feedback in the mini-CEX encounter. The present study is aimed to explore the feasibility of tutor-demonstrated feedback (TDF) in mini-CEX encounters and the resultant educational impact. Most mini-CEX feasibility studies focus on completion rates or user satisfaction ( Pelgrim *et al*., 2011). A minimum of four mini-CEX encounters per resident during one year is generally thought to be the number needed to document clinical competency ( Norcini *et al*., 1995; Alves de Lima *et al*., 2007).

The effect on learning by educational interventions is difficult to assess ( Barr *et al*., 2000) and it may be particularly so for the assessment of clinical competency e.g. by the mini-CEX. The reaction of the learners is commonly measured by their satisfaction (Level 1 of Barr’s adaptation of Kirkpatrick’s four-level evaluation model). Beyond that basic level of learning outcomes, there are increasingly higher levels of criteria required to assess the effect on learning like modification of attitudes/perceptions (Level 2a), acquisition of knowledge/skills (Level 2b), and change in behavior of the learners (Level 3) and ultimately the change in organizational practice (Level 4a) and benefits to patients/clients (Level 4b). Lorwald *et al*., (2018) reported some evidence of acquisition of knowledge/skills (Level 2b) from mini-CEX encounters by undergraduate trainees. In the case of learning by residents from mini-CEX encounters, however, there is only evidence based on the satisfaction of participants (Level 1) ( Miller and Archer, 2010).

Observation has been central to clinical practice since the early 19 ^th^ century ( Boudreau, Cassell and Fuks, 2008) and contributes to the learning of a wide variety of tasks as evidenced by the ample literature in different fields like education and training, behavioral, and neuro-imaging ( Rohbanfard and Proteau, 2011; Andrieux and Proteau, 2013; Lago-Rodriguez *et al*., 2014; Badets, Boutin and Michelet, 2018). Observing an expert perform provides the trainee with a clear standard for performing the task and promotes effective learning ( Rohbanfard and Proteau, 2011; Andrieux and Proteau, 2016). We decided to use the observation of performance by a faculty member as the TDF in the context of the mini-CEX. We used a group of trainees at the same level and asked them to peer assess in a mini-CEX encounter. Apart from the time taken for each encounter, we studied the differences in their assessment and satisfaction ratings before and after the TDF. There is little data regarding the number of peers required for reliable ratings of trainees in the mini-CEX encounters. In a study using peer global performance ratings to evaluate physician performance in areas such as clinical skills, humanistic qualities, and communication skills based on repeated observations, Ramsey *et al*., (1993) concluded that ratings from 11 peer physicians are required to achieve a reliability co-efficient of 0.70.

## Methods

### Study design and ethics

We conducted the study in the medical department of Bir Hospital, a 500 bed tertiary care government hospital, affiliated to the National Academy of Medical Sciences in Kathmandu, Nepal. There were thirteen final-year residents in the internal medicine program. We discussed the study with the residents and offered them voluntary participation which they all accepted. In each mini-CEX session a resident clinically assessed a patient in the presence of a tutor and peers and presented their findings. Later the tutor demonstrated the appropriate clinical behaviour for feedback on any performance gaps of the resident. The peers rated the performance of the resident before and after the TDF.

The residents were informed about the details of the mini-CEX including their right to withdraw at any time and that the mini-CEXs ratings would not impact on their summative assessment and the results would be analyzed after the completion of their training. We maintained the anonymity of the ratings by using code numbers instead of names. For analysis, each mini-CEX session was identified by the participant code number and serial number of the case.

Another challenge in the study was to mitigate the experimenter-expectancy effect, i.e. when the experimenter’s expectancies regarding the results bias the research outcome ( Sackett, 1979). If similar checklists were used before and after the TDF, the residents might think that any differences between the two ratings were being studied. In the mini-CEX forms used before the TDF, the terminologies were similar to that of the American Board of Internal Medicine, i.e. medical interviewing skills, physical examination skills, humanistic qualities/professionalism, clinical judgment, counseling skills, organization/efficiency and overall clinical competence ( ABIM, 2020). The terminologies used after the TDF were modified and similar to those used in other standardized observed clinical assessment tools e.g. complaints and history of the patient; physical examination method; examination findings; differential diagnoses, appropriate diagnosis and/problem lists; plan; concerns of and/or education of the patient, and presentation skills ( MRCP, 2017; ABIM, 2020; CPSP, 2020). A nine point rating scale was used to assess the performance of the resident ( ABIM, 2020) and was similar for both forms used before and after TDF. Satisfaction level with the mini-CEX was measured in both forms with the nine point rating scale ( ABIM, 2020). In each mini-CEX encounter the time measured related to the performance of the resident and the TDF.

It was also important to keep the mini-CEX encounters appropriate to the skills level for final-year medical residents in order to hold their attention and to increase the likelihood of impacting on their learning ( Lorwald *et al*., 2018). In the routine outpatient and emergency clinics, the final-year medical residents regularly assess patients usually within 15 to 20 minutes. We decided to use a similar format. There was no prepared case scenario; the resident had to elicit an appropriate history, carry out the relevant clinical examinations and formulate diagnosis and management plan in the presence of the tutor and peers. The resident could ask the patient any question and had to respond to questions from the patient.

The first author acted as the tutor for all the mini-CEX encounters in the study. Before each encounter, he explained the purpose of the session to the patients and made it clear that no individual details would be recorded. Before commencement of the study, ethical approval was granted from the Institutional Review Board of the National Academy of Medical Sciences, Bir Hospital (reference number: 197/2070/71). Subsequently it was endorsed by the University Research Ethics Committee of University of Dundee. Prior to starting the mini-CEX sessions, we conducted training of the residents on the mini-CEX process for two hours each day over three days similar as recommended ( Cook *et al*., 2009). The study took ten months to complete.

### The mini-CEX procedure

During each mini-CEX encounter a resident conducted a focused history and examination of an unfamiliar patient in the presence of the tutor and peers and summarized the findings. The peers rated the seven parameters relating to performance and the satisfaction level. They also recorded the time required to observe the performance. Next the tutor outlined any performance gaps and any aspects requiring clarification and then demonstrated the appropriate clinical performance and patient-findings. The tutor also addressed any concerns of the patient. Later the tutor further discussed any performance gaps and future learning plans with the resident. Peers again rated the earlier performance of the resident in the form designed for use after the TDF and noted the TDF time. The ratings and the discussions were carried out away from the bedside. The encounters with the first cases by each of the participants were completed one by one, and then the cycle of encounters with the second case by each resident was started and so on till the total six encounters were completed.

### Analysis

There were 1772 peer-assessment ratings in total from the two mini-CEX forms used before and after the TDF. Statistical differences were calculated using SPSS 17. Mean differences were analyzed by t tests and by Analysis of variance (ANOVA). Internal consistency of peer-assessment ratings was measured using Cronbach’s coefficient alpha.

## Results/Analysis

All 13 final year residents completed six cases, with a total of 78 mini-CEX encounters over a one year period. The mean interval between the mini-CEX presentations was 3.8 days and the mean time taken to complete each of the six cases by all participants was 48.5 days. In each of 78 mini-CEX encounters, there were two mini-CEX forms to be rated by each of the 12 residents. Due to the occasional absence of one or two participants, 1772 peer-ratings out of an expected 1872 were completed. The overall response rate for peer assessment was 94.7%. The reliability coefficients of the peer-assessment ratings before the TDF were .87, .89, .93, .92, .92, and .77 and .89, .84, .89, .92, .92, and .83 after the TDF.

### Peer-assessment and satisfaction ratings

Table 1 shows the means of the peer-assessment ratings (PARs) across the seven parameters before and after the TDF for each of the six cases. The PARs decreased significantly in each of the six cases after the TDF. The peers rated the same earlier performance more negatively after the TDF than before TDF. The mean peer-satisfaction ratings, however, increased significantly in each of the six cases after the TDF.

**Table 1.** Mean (SD) peer-assessment and -satisfaction ratings before and after the tutor-demonstrated feedback (TDF).

Peer-assessment ratings						
	Case 1	Case 2	Case 3	Case 4	Case 5	Case 6
Before TDF	4.39 (0.56)	3.96 (0.47)	3.74 (0.53)	3.83 (0.59)	4.01 (0.56)	4.02 (0.24)
After TDF	3.84 (0.56)	3.50 (0.32)	3.47 (0.49)	3.51 (0.56)	3.67 (0.54)	3.74 (0.31)
P-value	<0.001	<0.001	<0.001	<0.001	<0.001	<0.001
**Peer-satisfaction ratings**						
	**Case** **1**	**Case** **2**	**Case** **3**	**Case** **4**	**Case** **5**	**Case** **6**
Before TDF	6.39 (0.79)	6.45 (0.47)	6.71 (0.40)	7.10 (0.50)	7.19 (0.27)	7.24 (0.22)
After TDF	6.62 (0.75)	6.77 (0.43)	7.45 (0.42)	7.82 (0.38)	7.71 (0.21)	7.73 (23)
P-value	<0.05	<0.05	<0.001	<0.001	<0.001	<0.001

### Duration of the mini-CEX encounters

The mean (SD) observation times for each case were 23 (6.81), 29 (5.18), 27.31 (5.49), 20.77 (5.36), 17.23 (6.04), and 11.85 (3.31) minutes. The means (SD) of the total times of encounters for each of the six cases were 41.85 (6.07), 50.19 (5.37), 53.62 (6.97), 40.62 (9.62), 31.92 (7.58), and 23.85 (4.69). As the participants performed each mini-CEX encounter with the TDF, the mean observation time decreased from 26 (6.67) in the initial two cases to 14.54 (5.51) minutes in the last two (p<0.001). Similarly the mean (SD) total time for the mini-CEX encounters decreased from 46.02 (7.04) in the initial two cases to 27.89 (7.42) minutes in the final two (p<0.001).

## Discussion

The duration of the mini-CEX encounters with the TDF is fairly similar to that reported in other studies ( Norcini *et al*., 1995; Norcini *et al*., 2003; Hatala *et al*., 2006; Wilkinson *et al*., 2008; Davies *et al*., 2009), though the tutor-demonstration has added extra time. The duration of the mini-CEX encounters reported in the literature includes only the observation and the verbal and/or written feedback without tutor-demonstration time. The mean (±SD or range) or median duration in minutes of mini-CEX encounters among post-graduate trainees in different general medical specialties reported is as following:

• residents total time 31.5 (±19.3) ( Norcini *et al*., 1995);
• residents observation 18 (±12.1) and feedback 7.6 (±5.3) ( Norcini *et al*., 2003);
• residents observation 29 (26-31) and feedback 9 (5-13) ( Hatala *et al*., 2006);
• foundation doctors observation 15 and feedback 10 ( Davies *et al*., 2009);
• specialist-registrars observation 18.5 (1-90) and feedback 6.8 (1 - 75) ( Wilkinson *et al*., 2008).

Each participant completed six mini-CEX encounters with the TDF which is similar to that in other studies of the mini-CEX (an average 3 to 7 encounters per trainee) ( Norcini *et al*., 1995; Norcini *et al*., 2003; Hatala *et al*., 2006; Davies *et al*., 2009). The peer-assessment ratings, both before and after the TDF, are relatively low unlike that reported in the literature. The reliability coefficients of the peer-assessment ratings (ranging from.77 to .93) are similar to those in previous studies with ratings made for different patients by different assessors, e.g. .65 to.81 ( Norcini *et al*.,1995), .90 ( Durning *et al*., 2002) and .74 ( Hatala *et al*., 2006). Furthermore, peer assessment is shown to be reliable when the focus is specific ( Mavis *et al*., 2002). Though the mini-CEX and other similar instruments typically use global assessment scales ( Pelgrim *et al*., 2011), with the inclusion of tutor-demonstration of appropriate performance as the explicit standard, the focus of peer-ratings in our study should be more clear and specific and thus likely to be more accurate. Moreover the participants knew that the results would be analyzed by their code numbers only after the completion of their training and their evaluations would not have any impact on their final grade and thus they could freely assess their colleagues’ performance without concerns. There was no reason to inflate their ratings as happened when the exercise had greater implications for trainees ( Hawkins *et al*., 2010; Mitchell *et al*., 2011). The mini-CEX is also conventionally trainee-centered where the trainees choose the patients and assessors and the differences in case difficulty and rater stringency may also impact on the fairness of grading outcomes ( Hawkins *et al*., 2010; Mitchell *et al*., 2011). In the present study the residents encountered unfamiliar patients which may have caused lower peer-assessment ratings. Similarly the participants assessed the patients in front of their peers which could also have affected their performance.

The feasibility of conducting TDF in the mini-CEX is supported by the high completion rate for the various encounters and the increase in peer-satisfaction after TDF. This increase in peer-satisfaction ratings after TDF indicates learner reaction - the Level l educational impact on Barr’s adaptation of Kirkpatrick’s four-level evaluation model ( Barr *et al*., 2000) - due to the TDF. Though peers were more satisfied after the TDF their ratings of the same performance in the mini-CEX encounter decreased after the TDF compared to that before. The decrease in peer-assessment ratings after TDF could be due to the modification of attitudes/perception (Level 2a) of and the acquisition of knowledge/skill (Level 2b) by the participants as a result of the TDF. The observation of performance by an expert has been shown to promote effective learning ( Rohbanfard and Proteau, 2011; Andrieux and Proteau, 2013; Lago-Rodriguez *et al*., 2014; Badets, Boutin and Michelet, 2018). The educational impact on the participants is further indicated by the decrease in the observation time in the final two cases as compared to that in the initial two. This decrease indicates an educational impact at Level 2a, Level 2b, and even Level 3 (i.e. change in the behavior) as the participants successively performed in the mini-CEX encounters with TDF. Observation of performance by experts followed by physical practice is an effective way of learning ( Rohbanfard and Proteau, 2011; Andrieux and Proteau, 2016).

There is little evidence of positive educational outcomes on residents beyond satisfaction level in the mini-CEX literature (without TDF) ( Miller and Archer, 2010; Pelgrim *et al*., 2011). The TDF could be the missing link for the learning of trainees in the complex situation of clinical medicine where the clinical picture and findings of each individual patient are unique. A mixed observation schedule of performance by novice and expert followed by physical practice results in higher quality learning along with long-term retention ( Rohbanfard and Proteau, 2011; Andrieux and Proteau, 2013). The observation of performance by an expert provides an accurate template for performing the task and also allows trainees to detect and correct errors ( Rohbanfard and Proteau, 2011; Andrieux and Proteau, 2013). In our study, trainees had also no difficulty in recognizing themselves as novices and the tutor as an expert and in each mini-CEX encounter the tutor also outlined any performance gaps before demonstrating the appropriate performance. Such information about what they are to see (feedforward and observation) aids learning more than when this information is provided afterwards (as observation and feedback) ( Andrieux and Proteau, 2016).

The decrease in the observation time of the mini-CEX encounters has never been reported in the literature ( Norcini *et al*., 1995; Norcini *et al*., 2003; Hatala *et al*., 2006; Wilkinson *et al*., 2008; Davies *et al*., 2009; Hill *et al*., 2009; Brazil *et al*., 2012). This may be due to the use of different foci in the mini-CEX encounters. These are usually analyzed together which makes it difficult to compare the results or duration for any particular foci. As there are different foci possible in the mini-CEX, each foci or examination of a particular body system may be used only once or twice in the studies. Thus the duration of the observation may just be representing the duration of the initial encounters in various foci. Apart the possible educational impact on the participants by inclusion of TDF, the use of a single focus of integrated clinical assessment in all the mini-CEX encounters in the current study may have made it possible to observe the improvement in the performance of the participants and hence a decrease in the duration of the presentation in the later performances.

### Strengths and limitations

TDF in the mini-CEX encounter is a novel concept. The study had a unique design to indicate possible changes in the assessment and satisfaction ratings made before and after TDF in a single mini-CEX encounter.

This was a single institution study with a single cohort of final year residents, though there were adequate numbers of encounters, raters and peer ratings. Similarly, the TDF was carried out in all mini-CEX encounters by a single tutor. One possible confounding factor may be the use of unfamiliar patients in the mini-CEX encounter. Such an encounter is known to improve the learning ( Meagher and McLeod, 2001; Burch *et al*., 2006). The other possible confounding factor in the study could be the difference in terminologies of the seven parameters in the two mini-CEX tools. However the terminologies in the form used before the TDF were similar to that of the ABIM mini-CEX tool ( ABIM, 2020) and those in the second form used were similar to others used in standardized observed clinical assessment tools ( MRCP, 2017; ABIM, 2020; CPSP, 2020).

### Future directions for research

One area requiring further research is the feasibility of conducting the mini-CEX with TDF in different settings. The mini-CEX encounter could also be conducted with unfamiliar patients and different foci and among groups of trainees. There is some evidence of the educational impact of the mini-CEX compared medical students’ performance with their summative clinical results ( Lorwald *et al*., 2018). There is a need for similar research using residents. The Practical Assessment of Clinical Examination Skills (PACES) is used to assess medical residents ( MRCP, 2017) and has similar characteristics to the mini-CEX ( Table 2).

**Table 2.** Similar characteristics of the mini-clinical evaluation exercise (mini-CEX) and PACES * that can be used to assess educational impact after tutor-demonstrated feedback.

Characteristics	Mini-CEX (ABIM, 2020)	PACES * (MRCP, 2017)
Observation of the performance	• Yes	• Yes
Duration of each encounter	• Each about 10 to 20 minutes	• Each encounter station 10 or 20 minutes
Sampling	• Multiple with different examiners and patient encounters	• Multiple with total ten examiners and eight patient encounters assessing various clinical foci
Focus of encounter	• Data gathering • Diagnosis • Therapy and/or • Counseling	• History taking • Examination of four major body systems • Integrated clinical assessment (as brief clinical consultation) • Communication skills and ethics
Feedback: Timing and method	• After rating • Immediate verbal and/or written	• After rating • For all candidates: Marking results with the total of their marks by skill and encounter (called ‘feedback’) with the candidates encouraged to share the information with their educational supervisor • For those who perform very poorly: Structured letter (called ‘counseling’) compiled from the handwritten comments made by examiners on mark sheets

* PACES: Practical Assessment of Clinical Examination Skills, the summative clinical assessment of the Membership of Royal College of Physicians (MRCP) in the standardized setting

## Conclusion

The study demonstrates the feasibility and educational impact of the TDF in the mini-CEX. Further research needs to determine whether the findings in our study can be generalized to different settings and levels of trainees. The major strength of the mini-CEX is its brevity exposing the trainees to a range of clinical cases. TDF has the potential for effectively using the mini-CEX in the clinical field where the presentations and findings of each individual patient are unique.

## Take Home Messages

• TDF makes effective use of the mini-CEX as a supervised learning event and formative assessment.
• TDF in the mini-CEX has the potential to improve trainee performance in similar type clinical assessments e.g. PACES.
• It is feasible to use unfamiliar patients in mini-CEX encounters for training residents.
• Learning by observation of performance by novice and expert can be utilized for training of students in small groups.

## Notes On Contributors

**Prof M. D. Bhattarai**, MBBS, MD (India), DTCE (Japan), MRCP (UK), MMEd (Dundee) (ORCID: https://orcid.org/0000-0003-0872-1446) was a professor of medicine and coordinator of postgraduate medical training programmes in Bir Hospital, National Academy of Medical Sciences, Kathmandu where the study was conducted and since three years he is now a professor of medicine and faculty of postgraduate medical training programmes in Chitwan Medical College, Bharatpur, Nepal.

**Dr S. McAleer**, BSc, D. Phil is the Programme Director, Centre for Medical Education, University of Dundee, Scotland.
