# Supplementary material for: Tutor-demonstrated feedback in the mini-clinical evaluation exercise
Source: MedEdPublish (2016). 2020 Jun 23;9:130. [Version 1] doi: 10.15694/mep.2020.000130.1 (PMC10702658; doi:10.15694/mep.2020.000130.1)
Supplement: Peer review history

# Reviewer report: Reviewer response for version 1

**DOI:** 10.21956/mep.20045.r30705

**Published:** 2020-10-23

**Authors:** Ken Masters

**Affiliations:** 1 Sultan Qaboos University

This review has been migrated. The reviewer awarded 4 stars out of 5 An interesting paper on the impact of tutor-demonstrated feedback in the mini-CEX. The background establishes that, although the mini-CEX is widely used, there is a lack of evidence indicating the impact of the tutor feedback on the resident performing the mini-CEX, and the effect is commonly measured through learner satisfaction (Kirkpatrick level 1).This study aimed at measuring changes in peer evaluations and satisfaction before and after a tutor had demonstrated the correct process, has been well-constructed, and performed over a period of 10 months. Although the study is small, it is certainly reasonably conclusive, is a useful contribution to the field, and helps to shed light on the value of the min-CEX. I look forward to papers detailing larger studies, perhaps including qualitative data from interviews and/or focus groups, in which the various variables are investigated in more detail.

Reviewer Expertise:

NA

No decision status is available

# Reviewer report: Reviewer response for version 1

**DOI:** 10.21956/mep.20045.r30703

**Published:** 2020-06-25

**Authors:** Nandalal Gunaratne

**Affiliations:** 1 Faculty of Medicine, Wayamba University of Sri Lanka

This review has been migrated. The reviewer awarded 3 stars out of 5 A small study on a complex area. Perhaps it is continued and a further evaluation will be forthcoming in the future.

Reviewer Expertise:

NA

No decision status is available

# Reviewer report: Reviewer response for version 1

**DOI:** 10.21956/mep.20045.r30704

**Published:** 2020-06-25

**Authors:** Balakrishnan Nair

**Affiliations:** 1 Centre for Medical Professional Development and University of Newcastle

This review has been migrated. The reviewer awarded 5 stars out of 5 This is an interesting paper on minicex . It is a well studied tool for formative assessment and widely used in programmatic assessments. The major advantage of this assessment is the built in immediate constructive feedback. So this research paper on tutor demonstrated feedback expands our knowledge . The process had educational impact on the peers (learners) on the clinical issue, process of mini cex , and feedback. Hopefully these learners will be better clinicians and teachers in the future.

Reviewer Expertise:

NA

No decision status is available
